# Visual Diet versus Associative Learning as Mechanisms of Change in Body Size Preferences

**DOI:** 10.1371/journal.pone.0048691

**Published:** 2012-11-07

**Authors:** Lynda G. Boothroyd, Martin J. Tovée, Thomas V. Pollet

**Affiliations:** 1 Department of Psychology, Durham University, Durham, UK; 2 Institute of Neuroscience, University of Newcastle, Newcastle Upon Tyne, United Kingdom; 3 Department of Social and Organizational Psychology, VU University Amsterdam, Amsterdam, The Netherlands; University of Cordoba, Spain

## Abstract

Systematic differences between populations in their preferences for body size may arise as a result of an adaptive ‘prepared learning’ mechanism, whereby cues to health or status in the local population are internalized and affect body preferences. Alternatively, differences between populations may reflect their ‘visual diet’ as a cognitive byproduct of mere exposure. Here we test the relative importance of these two explanations for variation in body preferences. Two studies were conducted where female observers were exposed to pictures of high or low BMI women which were either aspirational (healthy, attractive models in high status clothes) or non-aspirational (eating disordered patients in grey leotards), or to combinations thereof, in order to manipulate their body-weight preferences which were tested at baseline and at post–test. Overall, results showed good support for visual diet effects (seeing a string of small or large bodies resulted in a change from pre- to post-test whether the bodies were aspirational or not) and also some support for the associative learning explanation (exposure to aspirational images of overweight women induced a towards preferring larger bodies, even when accompanied by equal exposure to lower weight bodies in the non-aspirational category). Thus, both influences may act in parallel.

## Introduction

In the early days of Evolutionary Psychology, much attention was given towards hypotheses that physical attraction may have certain universals across all cultures, due to selection for efficient mate choice. While this approach was successful with certain facial traits [1], [2] and there was early enthusiasm for female waist-hip ratio (e.g. [3]; though cf [4], [5]), it has always been patently inadequate when considering body weight preferences. Despite the pervasive nature of a thin-ideal in the Western world, samples from developing countries have repeatedly shown preferences for heavier women than samples in the West [6]–[10]. Furthermore, variation exists in the West over time, with thinner bodies being currently more in vogue than in earlier decades or centuries [11]–[13]. Importantly, there is evidence which shows that this variation may not be due to populations living in marginal conditions evolving a genetically underpinned preference for larger bodies than populations in more nutritionally rich environments: not only are preferences variable within Europe in a manner contrary to such a hypothesis [14], but it has been shown that there is large variability within the same ethnic group [7], [6]. Moreover Tovée and colleagues [15] found that migrants who have moved from South Africa to Britain have body size preferences which are intermediate between those of non-migrants in South Africa and Afro-Caribbean native Britons. The psychological mechanisms which underpin this potential change in individuals’ preferences, however, remain unstudied. Here we consider two possible alternative mechanisms: firstly that an individual’s ‘visual diet’ induces changes as a by-product of cognitive functioning, and secondly, the role of associative learning which may involve evolved learning biases.

The ‘visual diet’ mechanism represents a cognitive adaptation effect, where changes in preferences are induced by visual exposure to a certain variety of a given stimulus. There is a great deal of evidence that faces are neurally represented as points within a multidimensional ‘face space’ and that this space is flexible and can be ‘trained’ on artificial stimuli [16], [17]. After being shown a number of faces all adjusted in some common way, observers then perceive objectively neutral/average faces as showing the opposing effect. For instance after viewing smiling faces, neutral faces are seen as frowning. However, observers *prefer* faces manipulated in the same direction as those they have been trained on (i.e. which resemble their new mental ‘average’) [16], [42]. There is some preliminary evidence that this same effect may occur in body preferences. Winkler & Rhodes [18] showed observers images of black and white, headless bodies which had been horizontally stretched or compressed. At post-test, observers preferred a body size which was shifted in the same direction as the bodies they had viewed in the training phase. However, this involves a simple, unidimensional warping, which will clearly be susceptible to lower-level visual adaptation. As real individuals change body weight however, the appearance of greater/lesser weight is a composite of various topographical changes [19], [20], which may be less likely to produce low level adaptations. It is therefore essential that visual diet effects are tested with naturally varying stimuli.

The second potential mechanism is associative learning, whereby observers determine the relationship between body size and adaptively relevant traits, specifically status and health. Within Western media and culture, thinness is treated in an overwhelmingly positive manner [13], and by comparison being over-weight is stigmatized [21]. Body mass is also socioeconomically variable, with higher SES groups showing lower levels of obesity and lower BMIs in Western societies [22], [23]. Furthermore, in the West the abundance of nutritional resources means that almost no one is chronically malnourished. In contrast, many individuals suffer from health problems associated with obesity, and public health campaigns focus on the health benefits of lower (normal) weight (e.g. UK: Change4Life; US: Let’s Move). Thus, to Westernized individuals, thinness is associated with high socioeconomic status, high prestige (in terms of others’ approval) and better health.

On the other hand, in nutritionally stressed environments, body fat is believed to be an indicator of wealth and prosperity [24], with obesity as a symbol of economic success, femininity, and sexual capacity [25], [26]. Additionally in developing countries like South Africa, there are long-standing problems with infectious diseases, and the health consequences linked to these diseases include weight loss, and this is reflected in the perception that a lower body mass may signal potential parasitic infection or disease [27], [28]. This trend is exacerbated in countries with high prevalence of HIV/AIDS, where a potential cue to HIV infection is low body weight (“Wasting Syndrome”; [29], [30]). For both British and South African observers, perceptions of health and attractiveness are highly correlated, and it seems that what is thought to be healthy is also thought to be attractive [31]. So concerns about disease which are linked to BMI are likely to have a strong effect. When individuals migrate to the West, or become Westernized through media influence, they may simply be learning a different set of associations between weight and status/wealth/health for a novel environment. Indeed there is qualitative evidence from cultural studies of body image that after prolonged exposure to Western television, young girls may increasingly explicitly regard thinness as aspirational [32]. Furthermore, exposure to images of plus-size models (with faces obscured) is associated with an increase in preferences for more adipose faces, suggesting some higher level associative learning is taking place [33].

Here we attempt to distinguish between these two potential learning mechanisms using experimental methods to mimic the changes in visual/cultural information experienced by migrants. Across all our studies, participants are assessed for their preferences for thinner vs. heavier bodies, given a manipulation, and then retested on their body preferences. Under the visual diet hypothesis, exposing viewers to a series of thinner bodies should increase the degree to which they favor thinness, while exposing them to a series of larger bodies should *decrease* the degree to which they favor thinness. Importantly, this effect should exist regardless of whether the bodies presented are neutral or positive. Under the associative learning hypothesis, however, only the pairing of thin bodies with a positive valence, such as cues to status or health, should produce a shift in preferences to thinner bodies, while only the pairing of larger bodies with a positive valence should produce a shift to preferring larger bodies.

In Study 1, participants viewed a series of *either* slim *or* overweight bodies which were e*ither* generally aspirational in nature (beauty queens in high status evening wear, or catalogue models), *or* non-aspirational (under- or over-weight women standing in grey leotards). Thus, while the aspirational conditions allow both visual diet and associative learning to affect perceptions, any change in preferences in the non-aspirational conditions could only be due to visual diet effects. Finally, in Study 2, participants viewed a combination of thin and fat bodies with one weight group aspirational and the other not, in order to test an associative learning mechanism with visual diet effects ruled out.

Importantly, our perceptions of ideal body size are closely linked to our own body image and body shape dissatisfaction [34]–[36]. Given that in Western populations, concerns with one’s own weight and shape are endemic and exist from an increasingly early age [37], we also considered it essential to factor one’s own body image concerns into our design.

## Study 1

### Ethics

Ethical approval was obtained from the Durham University Department of Psychology ethics committee. Participants read the study information and as the study was conducted remotely online (for both student and nonstudent participants) consent was indicated by pressing the button to start the study. Participants were given information on eating disorder support organisations at the close of the study.

### Materials and Methods

#### Participants

Fifty-seven women (mean age 26.4 years, s.d. 9.9) who reported their sexual orientation as mostly/exclusively heterosexual (scoring 5–7 out of 7, where 7 = exclusively heterosexual and 1 = exclusively homosexual) were recruited from the departmental participant pool and from a UK-based internet forum for women. Participants were told the experiment was on ‘how we perceive bodies’ and that they would compare different kinds of bodies in different ways; the study’s precise aims and hypotheses were not revealed until the end. Participants self-allocated into the four conditions on the basis of their birthday (e.g. 1^st^ to 7^th^ of month = Condition A), and this allocation was counterbalanced.

#### Questionnaire data

Before starting the study, participants were asked to complete the short version B of the Body Shape Questionnaire (BSQ, [38]). The BSQ asks participants to estimate the frequency in the preceding four weeks that they have engaged in certain body-dissatisfaction behaviors or cognitions, and higher scores reflect greater levels of dissatisfaction. Responses were averaged across all questions and average scores ranged from 1.0 to 5.88 (maximum score is 6) with a mean of 2.6. Participants were then split into ‘high’ (more than 2.6) and ‘low’ (less than 2.6) body satisfaction groups. Due to a computer error, BSQ scores were missing for 13 participants in Condition A.

#### Pre-test preference task

**Figure 1:**
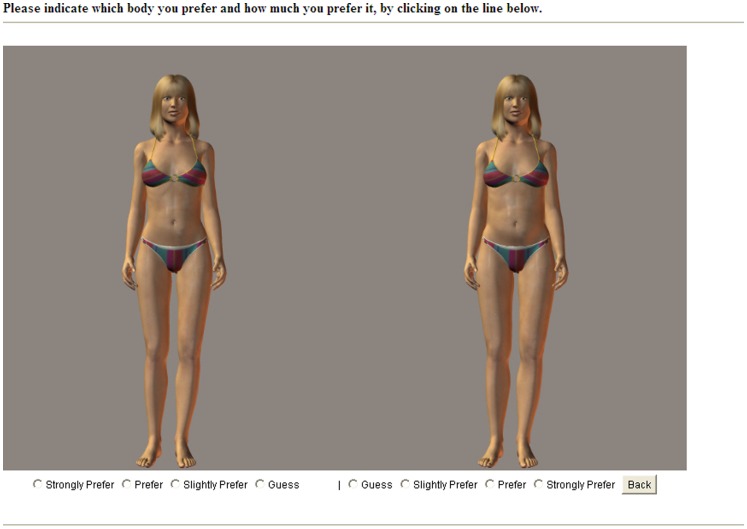
Example frame from the body size preference task.

Participants were told that the first phase of the experiment was to ‘compare CGI bodies side by side’, and were given a body-size preference task. 10 female face-on bodies dressed in a bikini were produced using the Poser 6 3D modelling software package (Smith Micro Graphics) using the Victoria 4.0 (V4) female model (Daz3D.com), which allowed controlled manipulation of the size and shape of the bodies produced. These were altered using the V4 body morphs to mimic the effect of increasing body fat. To quantify the effect of this change, bodies were imported into the 3ds Max modeling package (usa.autodesk.com), and height and volume was measured. Assuming the bodies to represent women of average height (167.5 cm) with an average body density of 1.1 g/cm^3^ [39], we can then calculate an estimate of the models’ body weight and so their BMI. The bodies consisted of the same female ‘model’ but were altered to range in apparent body mass from 17.2 to 30.9 in 10 intervals. These were then presented in two interval pairs (i.e. Body 1 with Body 3, Body 2 with Body 4, Body 3 with Body 5, etc.) in a randomized order. For each pair, participants indicated which image they preferred and how much they preferred it, ranging from ‘guessing’ to ‘strongly prefer’ (data was recorded on an 8-point scale ranging from 0 [strongly prefer the larger body] to 7 [strongly prefer the thinner body]). Final scores for each pair thus represented the degree to which the participant preferred the thinner bodies, where 3.5 represented no preference either way. Scores were averaged across all trials to produce a total ‘preference for thinness’ score. A screen-shot of the preference task is shown in Figure 1.

#### Manipulation phase

During the manipulation phase, participants viewed a series of images of women. For Conditions A and B, stimuli were drawn from images of models and beauty queens. In Condition A, images consisted of 16 images of models from UK clothes retailers’ catalogues (*Next*, *New Look*), 10 Miss America finalists in evening wear and Miss England 2007 in swimwear. In Condition B, images consisted of 16 images from Plus Size clothes ranges’ catalogues (*New Look Plus*, *Evans*, *You*), 10 Miss Plus America finalists in evening wear, and the runner up of Miss England 2007 (who was a plus-size contestant) in swim wear. In each condition, images were presented twice, once in ‘’normal’ alignment, and once in mirror-opposite, giving a total of 52 trials in both conditions.

For Conditions C and D, stimuli were taken from a collection of photographs of women of known BMI, dressed in grey leotards, previously used in published papers [40], [41]. Condition C used seven with BMIs between 15 and 20. In order to increase the number of stimuli, the photographs were altered to add a border – four different versions were produced: light red, dark red, light purple and dark purple. Mirror image versions were then also produced, giving 56 trials in total. Condition D used seven images of women with BMIs between 25 and 30 and 56 final trials were created in an identical fashion.

Participants were told this phase of the study was looking at ‘sequential judgements of real images’. In order to keep attention focused on the stimuli, participants were asked to compare each image to the one they had seen before and indicate which was the more attractive (for the first trial they were told to compare it with the previously seen CGI images). As soon as the participant had indicated their relative preference for the image, the next image was automatically presented. Order of stimuli was fully randomized.

#### Post-test preference task

Immediately after the manipulation, participants were told they were completing ‘the second half of the CGI test’ and completed the same preference task as at pre-test. Pre- and post-test preferences for thinness scores were significantly correlated across participants (*r* = 0.686, *p*<0.001).

Following the post-test participants were thanked and debriefed.

### Results

**Figure 2:**
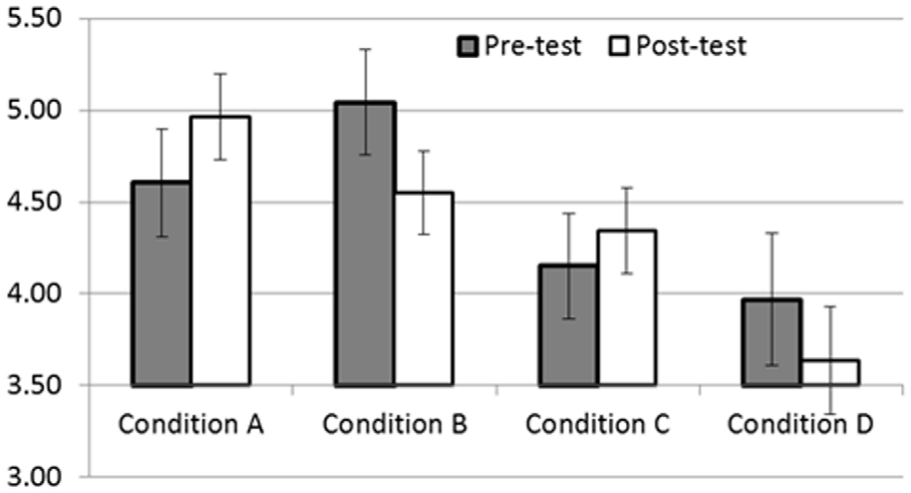
Mean ‘preference for thinness’ at pre- and post-test, for each condition in Study 1. Those in Conditions A and C were shown thin bodies during the manipulation, while those in Condition B and D were shown large bodies. Those in conditions A and B were shown aspirational models and those in conditions C and D were shown non-aspirational models.

Results were analysed using a mixed ANOVA design, where test phase (pre- or post-test) was a repeated measures variable, and model size (large or thin) and model type (aspirational or non-aspirational) were between group variables. As predicted by the visual diet hypothesis, there was a significant interaction between test phase and model size (*F*_1,52_ = 23.397, *p*<0.001, partial eta^2^ = 0.310) such that preference for thinness increased from pre- to post-test in those shown thin models during the manipulation phase, while preference for thinness decreased in those shown large models during the manipulation. In contrast to the associative learning hypothesis, this interaction was not qualified by a three way interaction between phase, model size and model type (*F*_1,52_ = 0.548, *p* = 0.5, partial eta^2^ = 0.009), meaning that the phase x model size interaction held for both aspiration and non-aspirational models. Apart from a marginal main effect of model type, such that those in the non-aspirational conditions preferred thinness more than those in the aspirational conditions (at pre and post-test), there were no other main effects of interactions (all *F*<1, all *p*>0.3). Furthermore, the inclusion of BSQ group as a between groups variable (which also reduced sample size) did not change the pattern of results and BSQ group did not interact with phase and model size, nor with phase, model size and model type. Means are given in Figure 2.

### Interim Discussion

This first study supported the visual diet hypothesis, in that presentation of a series of thin models during the manipulation phase had the effect of increasing participants’ preference for thinness, while presentation of large models had the opposite effect, for both aspirational and non-aspirational images. While the associative learning hypothesis would have predicted an effect for the aspirational images, it would have predicted no effect (or perhaps even a negative aversion effect) for the non-aspirational images of very over/underweight women.

However, it should be noted that this first study used a combination of images (e.g. some had borders and the number of trials was not matched between conditions). Although it seems unlikely that 52 versus 56 trials could make a difference, it may yet have led(?) to slightly stronger effects in the non-aspirational trials that would have been achieved otherwise and may thus mask any three-way interactions. Furthermore, although these data support the visual diet hypothesis they do not tell us whether visual diet is the only means by which change in weight preferences may arise. In order to further explore this issue we therefore conducted a second study the second study used conditions in which a visual diet effect would be impossible (equal numbers of large and thin model, of differing type) but where an associative learning effect may still arise. If these conditions fail to produce an effect on participants’ body preferences then it may be concluded that visual diet alone can explain the patterns seen, for instance, in Tovée et al’s Zulu migrants.

## Study 2

### Materials and Methods

#### Participants

69 female Psychology undergraduates aged 18 to 27 years were recruited from the department participant pool in part fulfillment of course requirements (mean age 19.36 years; s.d. 1.9). Participants were again all mostly or exclusively heterosexual. Invitation emails were sent to students in a randomized manner (6 students per day) and the first and third quartiles of the sample were directed to Condition E (final N = 37 after exclusion of 9 males and 6 women with incomplete questionnaire data), while the second and fourth quartiles of the sample were directed to Condition F (final N = 32).

#### Manipulation phase

All aspirational stimuli from Study 1 were given borders in an identical fashion to the non-aspirational stimuli such that all stimuli matched in this respect. In Condition E, participants were shown 40 images from Condition A (thin models) and 40 images from Condition D (large leotard bodies), while in Condition F participants were shown 40 images from Condition B (large models) and 40 images from Condition C (thin leotard bodies). Thus Condition E participants were being exposed to a thin = aspirational/large = non-aspirational association, while Condition F participants were exposed to large = aspirational/thin = non-aspirational, and all participants were viewing equal numbers of large and thin women. In order to further emphasise the difference between stimuli groups, participants were asked to separately rate how much they liked each image from 1 (not at all) to 7 (very much).

In all other respects, Study 2 was identical to Study 1. As before, pre- and post-test preferences for thinness scores were significantly correlated across participants (*r* = 0.666, *p*<0.001).

### Results

As participants were rating stimuli for how much they liked them, it was possible to compare stimulus groups for pleasantness in order to confirm whether the ‘aspirational’ models were indeed preferred to the ‘non-aspirational’ bodies. Using a two-way ANOVA with image as the unit of analysis, and model size and model type as between participants variables, it was found that there was a main effect of model size such that thinner models were ‘liked’ more (*F*_1,196_ = 159.974, *p*<0.001, partial eta^2^ = 0.449), and of model type, such that aspirational models were ‘liked’ more (*F*_1,196_ = 121.762, *p*<0.001, partial eta^2^ = 0.383). There was no interaction between the two (*F*_1,196_ = 1.568, *p* = 0.212, partial eta^2^ = 0.008). Between groups comparisons showed that thin aspirational models were liked significantly more than all other models (mean = 3.3, Tukey’s *p*<0.001); large non-aspirational models were liked significantly less than all other models (mean = 1.4, Tukey’s *p*<0.001); and thin non-aspirational models and large aspiration models did not differ from each other (means = 2.3 and 2.2 respectively, Tukey’s *p* = 0.665).

**Figure 3:**
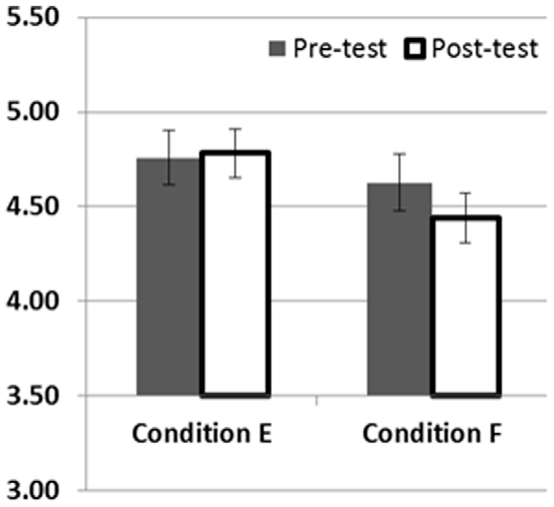
Mean ‘preference for thinness’ at pre- and post-test, for each condition in Study 2. Those in Condition E were shown a combination of thin aspirational bodies and large non-aspirational bodies during the manipulation phase, while those in Condition F were shown large aspirational bodies and thin non-aspirational bodies.

For the main analyses, as in Study 1, a mean-split (mean = 3.27) was performed on the BSQ scores and as all participants had full BSQ data it was included from the outset. A mixed ANOVA with phase as a repeated measures variable, and condition and BSQ group as between participants variables showed no significant interaction between phase and condition (*F*_1,74_ = 1.704, *p* = 0.196, partial eta^2^ = 0.023). However, the pattern of means did suggest that there was a difference in the predicted direction in Condition F. A further ANOVA was therefore conducted for Condition F only, with phase as a repeated measures variable, and BSQ group as a between participants variable. This showed a main effect of manipulation such that thinness preferences at post-test were significantly lower than at pre-test (*F*1,30 = 4.386, *p*<0.05, partial eta^2^ = 0.128; means are given in Figure 3). There was no interaction with BSQ group (*F*1,30 = 1.671, *p* = 0.206).

## Discussion

The purpose of the current study was to test different mechanisms by which the intra- individual change in body size preference potentially documented by Tovée and colleagues [15] could have arisen. We considered that such variation could arise due to a change in visual diet due to media saturation of low-BMI bodies, or could arise due to the internalization of an association between thinness and high status/health in the West. Overall, these results show that exposure images of overweight or low weight women can induce a change in the body size preferences of female observers, no matter whether those images are aspirational (i.e. evoking cues to health/status etc) or not. This shows strong support for the visual diet hypothesis of body preference change, i.e. that participants in Conditions A to D were potentially adjusting their perceptions of ‘average’ based upon recent visual experience regardless of any valence information accompanying that experience. These data support the findings of the adaptation research conducted on faces and Winkler & Rhodes’ [18] study with bodies, and show for the first time an adaptation-like effect on visual preferences with naturally varying stimuli.

Surprisingly, however, the effect of exposure to images of aspirational overweight women persists even where such images are paired with non-aspirational images of underweight women. That is to say, given that visual diet cannot explain such changes, those in Condition F must have been responding to the positive valence which accompanied the larger bodies (although it is notable that Condition F participants do not ‘like’ the larger bodies more than the thinner bodies, this equality is in itself a counter-cultural association). As such the results also show some support for the associative learning explanation of body preference change, and suggest that the Zulu migrants to the UK studied by Tovée and colleagues [15] may also have changed their preferences due to the positive associations with thinness in their new cultural environment as well as overall changes in visual diet.

It is important to consider, however, why there were no significant changes in Condition E which should have (according to the associative learning hypothesis) induced an increased preference for thinness. Given that our participants in Conditions E and F were all young British females, and several years younger than those in other conditions, it is highly likely that their environment is already particularly saturated with positive associations with thinness and that the ‘thin is good’ trials represented the participants’ pre-existing position. Indeed, in all conditions, and even after the larger bodies manipulations, all participants reported on average a preference for thinner-than average bodies (see graphs; where 3.5 represents no preference or an equal balance between preferring thinner bodies in some trials and larger bodies in the others). Thus, there was potentially limited scope for increasing thinness preferences particularly amongst a young student population. In contrast, however, the ‘fat is good’ trials represented a counter-normative association and was in fact far more analogous to the situation experienced by Zulus migrating to the UK who moved from an environment where fatness is positively valenced, to an environment where thinness is preferred. In that sense, it is these latter conditions which more accurately test the potential mechanisms at work in Tovée and colleagues’ Zulu participants.

In summary, we have reported data which suggest that amongst female observers, cross cultural and trans-historical variation in body weight preferences may reflect both the adaptation of internal prototypes to current visual norms and the internalisation by individuals of cues to status, prestige, or health which covary with body weight. In our stimuli, however, we have conflated status (clothes), health (e.g. slimness vs. emaciation) and general positive valence (facial attractiveness of models and pleasantness of setting). It is therefore essential that further research distinguishes between the different elements which may be driving the learning observed in Condition F. In addition, further research with more representative sample of females (especially for Conditions E and F) and particularly with male participants is also needed. Our results have general relevance to human perceptions and interactions outside a mating domain. However, given that men are more likely to see female bodies as potential mates, the relative importance of visual diet and associative learning to mate choice is an important further consideration.
